# Transitioning from pediatric to adult care and the HIV care continuum in Ghana: a retrospective study

**DOI:** 10.1186/s12913-021-06510-4

**Published:** 2021-05-17

**Authors:** Pearl Abaka, Jerry John Nutor

**Affiliations:** 1 Ghana College of Nurses and Midwives, Westlands, Accra, 233 Ghana; 2 Department of Family Health Care Nursing, School of Nursing, University of California San Francisco, San Francisco, California 94143 USA

**Keywords:** Adolescents, Healthcare transition, HIV, Adult clinic

## Abstract

**Background:**

In Sub-Saharan Africa, there are now a significant number of adolescents living with HIV (ALHIV), due to increased access to effective antiretroviral therapy. However, these adolescents are at high risk of dying during the transition to adult care due to various reasons, including lack of preparation for the transition and poor transition arrangements. More knowledge about this issue will lead to a better planned healthcare transition process and preparation for transition from pediatric care to adult care. The aim of this study was to explore the healthcare transitional experiences of ALHIV as they moved from pediatric to adult care.

**Methods:**

A descriptive exploratory qualitative study was conducted. Purposive sampling method was used to recruit adolescents between 12 and 19 years old. Saturation was realized by the 10th participant. Data were analyzed using thematic content analysis.

**Results:**

Four main themes emerged from the interview data: the transition process, factors facilitating the transition experience, challenges and coping mechanisms of the ALHIV during transition, and suggestions for improvement based on perceptions on the current transitioning approach. A key finding of this study was the sudden preparation for transition, linked to the absence of a structured transition protocol. Even though age was the main reason for transferring the participants from the pediatric to adult clinic, participants’ age did not influence whether they attended clinic appointment on their own or accompanied by a care provider; it was dependent on the availability of their parents or caregivers. Participants’ parents and adult family caregivers were also integrated into the transition process to some extent. We also found that most of the participants had good patient-provider relationship with their health care providers in both pediatric and adult clinics.

**Conclusion:**

Findings support the need to develop a structured healthcare transition policy and age-appropriate transition within the clinic environment. There is also a need for social and community support as ALHIV transition from pediatric to adult care.

## Introduction

Globally, the prevalence of HIV among adolescents remains high. According to the World Health Organization (WHO), there are approximately 1.8 million adolescents, between 10 and 19 years old, living with HIV in the world in 2019, and about 1.5 million (85%) of these adolescents are living in sub-Saharan Africa [1, 2]. Efforts to improve access to antiretroviral therapy (ART) have resulted in increased survival rates for adolescents living with HIV (ALHIV) [3]. It is estimated that about 25,000 ALHIV transition from pediatric to adult healthcare at some point [4]. It is also estimated that approximately 10% of these adolescents die during the period of transitioning to adult care due to various reasons, including lack of preparation or arrangements for transition [5, 6], lack of best practices or models of service [7], and fear of separation and stigma [8]. Poor transition from pediatric to adult care is also associated with loss to follow-up or poor retention in care and poor adherence to ART [9], which could explain the high death rates among ALHIV during the transition period from pediatric to adult care [5].

Transitioning from pediatric to adult healthcare is recognized and defined as the planned and purposeful movement of an adolescent from child-centered to adult-centered care [10]. Transitioning should be an active process that prepares and caters to the medical, psychosocial, and educational needs of adolescents as they prepare to move from pediatric to adult-oriented care. The age at which this transition should occur is not specified and can vary by country and culture. Regardless of the adolescent’s age, transition scheduling should address multi-factorial concerns about their cognitive development and mental health, medication adherence, sexuality and gender identity, reproductive status, socioeconomic and health insurance status, stigma and disclosure, the impact of a disrupted relationship with the pediatric health care provider (HCP), and communication [11]. Therefore, a multidisciplinary and developmental approach early in the process is needed for a successful care transition.

However, poor transitioning experiences among ALHIV have been widely reported [5, 9, 12]. These reports include lack of available healthcare transitioning clinics and poor preparation for the transition [13]. ALHIV often see the transition to adult clinic as a hostile process to disconnect their relationships with friends and HCPs at the pediatric and/or adolescent clinic, and sometimes act to protest the process [12]. In cases where an adolescent transition clinic does exist, the model of care was reported to be inadequate based on expectations, missing services regarding pre-transition care counselling and emotional preparation [14]. Various other challenges have also been reported, and include poor support for healthcare continuity, stigma from adults, unfavorable adult clinic appointment schedules, and crumbling of peer support networks [14]. Factors that can have a positive influence on the transition process include the adolescent’s level of maturity, knowing the kind of treatment they receive in addition to ART, the family receiving social support for their healthcare needs, trust in the family for HIV treatment, receiving transition counseling, financial security, having a ‘Case Manager’ for support during preparation for the transition process, and satisfaction with general preparation for the transition process [15–17].

Based on these prior studies, a well-planned transition process and adequate preparation of the adolescent can impact health outcomes. The issue of adolescent transition has received even greater attention since 2014 with the United Nations Programme on HIV/AIDS (UNAIDS) 90–90-90 global and national targets, particularly for the target aimed at ensuring that at least 90% of children on ART are retained and virally suppressed by 2020 (UNAIDS, 2014). This global initiative placed effective transitioning of ALHIV to adult care on the global policy agenda, resulting in increased demand for strategies to improve outcomes.

Ghana is among the nine countries in West and Central Africa that contributes to 90% of new pediatric HIV infections, with 40% of new infections occurring in children [18]. Similar to other countries in sub-Saharan Africa, Ghana has a significant number of ALHIV who need transition care because of the increased survival rates into adulthood. At the end 2017, Ghana had 28,000 children under 14 years of age, and 19,000 adolescents (10–19 years of age) living with HIV [2]. Ghana has made progress in addressing the many challenges faced in the HIV crisis, but gaps in pediatric HIV care remain associated with poor health outcomes and deaths [18]. Although transition care itself has been linked to health outcomes as these young clients age, not much is known about the transition process, practices and challenges. With no national guidelines for transitioning ALHIV from pediatric clinics to adult clinics health centers in Ghana, protocols vary from one health facility to another. Therefore, the purpose of this study is to provide much needed baseline information about adolescent experiences of transition to adult care. A better understanding of the adolescent’s perspective of challenges experienced during transitioning will serve to provide the HCP and other stakeholders with direction for strategies to enhancement the continuum of care for the ALHIV population and promote better health outcomes as young adults.

## Materials and methods

### Study design

This qualitative study was conducted using a descriptive exploratory qualitative design that involved performing in-depth interviews with ALHIV. This approach was considered more appropriate for this research because the experiences of ALHIV during transition from pediatrics to adult care has had limited research and is not a well-understood area of healthcare.

### Sample and setting

In order to obtain a wide range of experiences in the transition from pediatric to adult care, purposive sampling was used to select adolescents between 13 and 18 years of age who accessed services at a referral clinic site for the Accra Metropolis region. The HIV clinic is a referral center responsible for the registration and management of all HIV pediatric and adult patients. This clinic site was purposefully selected because healthcare transfer/transition is done for ALHIV seeking care at the facility. Transfers from the pediatric clinic to the adult clinic are done by 13 years of age.

Adolescents and their parent or guardian/caregiver were informed about the study by the attending nurse during a regular clinic visit. Potential participants who wanted to know more about the study were referred to a trained research assistant who explained the details of the study using the information sheet provided to the interested adolescent to read. After addressing questions about the study and consent form, potential participants and the guardian/caregiver signed the consent form. For participants who were not yet 18 years old, the parent or guardian voluntarily signed the parental/guardian consent form and the adolescent also assented and signed the child assent form. Permission was sought from participants and their parents/guardians for the interview to be audiotape recorded.

Participants were included in the study if they were between the ages of 13 and 18 years, tested positive for HIV, and had transitioned to the adult HIV clinic at least 1 month before data collection. We excluded adolescents who were seriously ill, defined as hospitalization.

### Interview guide

The in-depth interviews were conducted using a semi-structured interview guide designed by the authors and reviewed by two experts for content and flow of the questions. The guide was pilot-tested with three ALHIV from Tema General Hospital in the Greater Accra region and probes were added for flow, clarity and to fill in gaps. Section A of the interview guide consisted of demographic questions about participants and Section B consisted of open-ended questions and probing questions specific to the objectives of the study. The focused questions were on the healthcare transition experiences of ALHIV, facilitators, and their perceptions of or challenges/barriers during transition. Example of these questions included: “Please tell me how you moved from the child to adult HIV clinic.” “How are you coping with your new environment, healthcare providers and friends at the new clinic?” “What differences have you notice between the children and adult clinic?” The participants’ responses also determined the length, direction, and flow of the interview.

### Data collection

All ALHIV who met the inclusion criteria were offered the opportunity to participate. Those who consented were interviewed in one of the counselling rooms at the clinic with only the participant and the interviewer present. Although interpretation for local languages was provided for Ewe, Twi and Ga into English, all participants opted to be interviewed in English. Each interview lasted approximately 20 to 30 min. Probing questions were asked if answers provided by the participants were not clear to the researcher.

Participants’ non-verbal cues such as facial expressions, mannerisms, and gestures were documented in field notes. The environment and all sequence of events, including any interruptions during an interview, were also noted. Due to the sensitive nature of the interview, the services of a trained counsellor were provided in case of any emotional distress to the participants; none of the participants made use of this service. However, the interviewer referred all participants to the trained counsellor after the interview to assess their emotional state.

### Rigor and trustworthiness

The rigor of the results and the study was confirmed through credibility, transferability, dependability and confirmability [19]. Credibility was established by conducting this research in such a way that confidence in the truth of the data collected during the interview was evident, by structuring the interview guide to elicit accurate responses to questions through the use of strategies such as providing privacy and confirming responses if the interviewer was not clear about their answers. Purposive sampling was used to select the number of adolescent participants required who could openly share their experiences about living with HIV. During the interviews, the researcher repeated certain phrases or words expressed by the participants for clarification. In situations where the response was still unclear, participants were asked to confirm their response after the interview to verify the exact information disclosed. The transcript of each interview was independently coded and classified by the researcher, supervising professor and consulting professor. The results for each coded response were compared and any discrepancies were reviewed and amended by the authors.

Transferability is the extent to which the findings from the data can be transferred or generalized to other populations or settings. Transferability was enhanced by a thorough description of the design and methodology, the research environment in the Greater Accra Regional Hospital clinic, and the number of participants involved so that findings can be replicated by other researchers. Results of the study were shared with non-participants and feedback on the extent to which the results matched with their experiences indicated a high similarity between our findings and their experiences.

To achieve confirmability, the researcher conducted the study in an orderly manner, keeping field notes to describe events and processes pertaining to data collection, and ensuring that data analysis was factual and impartial. The researcher recorded and transcribed interviews verbatim to ensure that the findings truly reflected the perceptions of experiences of ALHIV.

To enhance dependability, or the stability of the data over time and over conditions [19] data analyses and codes were audited by an external reviewer. This inquiry audit also included relevant supporting documents and field notes to search for any inconsistencies.

### Ethical considerations

The study was conducted in accordance with the Declaration of Helsinki for research involving human subjects. Ethical approval for the study was obtained from the Ghana Health Service (GHS) Ethical Clearance Committee with reference number GHS-ERC-030/03/19. Permission was also sought from management through the Research Unit of the hospital clinic facility where the study was conducted. Confidentiality was ensured at all stages of the process. Informed consent was obtained from the participants before the interview. No minor was recruited without informed parental consent. In addition to informed parental consent, informed written assent was obtained from all ALHIV under 18 years of age prior to their participation. Participants were given snacks and 50 Ghana Cedis (10 USD equivalent) as compensation for their time.

### Data analysis

The interview data were analyzed using thematic content analysis based on the research objectives using NVivo software, version 12 (QSR International). While listening to the audiotape recordings, the researcher transcribed the contents verbatim into a word document immediately after each interview. Each transcript was checked for accuracy against the recording. For data immersion, the researcher read the transcript and listened to each recording several times to reach an overall understanding. To blind real identities, each transcript was labelled with a pseudonym and code number generated for the participant.

Data were kept in a narrative form that described the various nodes established in the coded thematic analyses. Conclusions were drawn from the nodes and child nodes were identified to represent the experiences of ALHIV transitioning from pediatric to adult clinic. Member-checking was also done by the researcher’s supervisor and consulting professor to ensure that the nodes represented the participants’ experiences. A number of discussions were held to ensure that the findings accurately represented responses from all ten participants.

## Results

The ALHIV participants interviewed for this study included five males and five females between 13 and 18 years of age. Their demographic characteristics are provided in Table 1. Four main themes and 13 sub-themes emerged from the interview data. The main themes and sub-themes are presented in Table 2.

**Table 1:** Socio-demographic characteristics of participants (*N* = 10)

Characteristic	Frequency (n)
**Sex**	
Males	5
Females	5
**Age**	
13	1
14	2
15	2
16	1
17	3
18	1
**Religion**	
Muslim	2
Christian	8
**Level of Education**	
Primary	1
Junior High	5
Senior High	4
**Ethnicity**	
Ashanti	4
Ga	2
Dagomba	2
Ewe	1
Fante	1
**Health Insurance**	
National Health Insurance	10
Other Health Insurance	0

**Table 2:** Themes and sub-themes

Theme	Sub-themes
Transition preparedness	● Pre-transition information ● Readiness for transition ● Duration of transition preparation ● Family caregiver involvement ● Coordination of care
Factors facilitating transition experience	● Child-specific factors ● Family support
Challenges and coping mechanisms during transition	● Challenges during transition ● Coping and adapting to challenges
Current transition approach and suggestions for improvement	● Perceptions about health during transition ● Perceptions about health care providers ● Perceived benefits of transition ● Suggestions for improving transition

### Theme 1: preparation for the transition process

The adolescents were asked how they were prepared for transition from the children’s clinic to the adult’s clinic, what information was given about the transition, who provided them with the information, and the extent to which they felt prepared for the transition. Five sub-themes emerged: pre-transition information including disclosure of HIV status, readiness for transition, duration of transition preparation, family caregiver involvement in the transition process, and coordination of care.

Participants indicated that most of the activities were directed by the HCP and could not describe any fixed structure or process that guided transition care, planning for the transition, coordination, integration or post transition care. When asked about the required meeting between the adolescent and HCP to create a transition plan to be followed, none of the adolescents in this study described such an opportunity. They responded that only that the HCP or caregiver informed them about the transition.

One recounted how she found herself at the adult clinic;

*“No one told me, I just followed my mum, I don’t know if someone told her or not. I am sure that I was prepared because I came with my mother and joined the adult clinic around 12 years old. I started taking the medicine at 2 years”* (P5)

Most participants could not describe the assessment process used by the HCP for transition readiness. There was no mention of using recommended readiness assessment tools. Some participants were verbally asked, with their age as the main determinant for transition. A participant explained as follows:

*“Well, they said I need to join them (the adult clinic) because I was above 12 years, so they asked if I can take my medicine by myself and I said yes. They also asked if I come regularly for checkup and I said, yes. So, they said I can go to the adult clinic, so when we came again, we didn’t go to the children clinic and we came here”* (P5).

#### Pre-transition information

Based on the interview data, there was no evidence to suggest the presence of a structured transition process. Most ALHIV were simply told to start going to the adult clinic. In some cases, a nurse accompanied them, yet the participant could not explain why. Surprisingly, some of the adolescents were transferred even before they were told their HIV status.

*“Maybe they informed my mother but as for me they did not inform me, so I just moved and joined because I didn’t even know about the sickness*” (P4)

“*The one who sent me there (to the adult clinic), Aunty M, she is a nurse who works there*” (P3)

“*I join the adult clinic at 13 years. The nurse brought us to the adult clinic. I wasn’t the only one, there were other adolescents. I don’t remember our number, but I remember they said we were old enough to join the adults now, so the nurse brought us”* (P7)

#### Readiness for transition

Generally, participants expressed satisfaction with transitioning from the pediatric to the adult clinic but had varied views about feeling ready for the transition. While some could not clearly express their readiness for the transition, others were not sure they were prepared for the adult clinic because they were not even sure what readiness entailed. The following quote supports this sub-theme:

*“Am not sure that I was prepared because I just came with my mother*” (P5)

In contrast, another participant expressed readiness to join the adult clinic because she was informed that she was of age by a HCP at a previous clinic appointment:

“*I was ready to join because the pharmacist told me earlier that I was grown enough to join the adult and myself I wanted and was ready to join”* (P6)

#### Duration of preparation for transition

The transition preparation time period was difficult to determine because the pediatric clinic did not have a structured transition protocol. For most participants, the transition was described as sudden or abrupt. Some mentioned that they moved to the adult clinic on the subsequent appointment the day they were told to start going to the adult clinic. Similar to what was voiced by others, this participant said:

“*One nurse told me about one month before I finally came to the adult clinic*” (P6)

#### Family caregiver involvement in the transition process

In response to an open-ended question about involvement of the caregiver in the transition process, most indicated that a family caregiver was involved. In some cases, the caregivers seemed to be more involved in the transition process than the adolescents themselves. Some adolescents acknowledged that their caregiver was informed about their transitioning.

The adolescents presented different thoughts about their caregiver’s involvement in the transition process. One adolescent mentioned that her grandmother was around when the HCP was informing her about going to the adult clinic:

“*Yes, my grandmother was there but it seems she was already aware because the doctor said she already told her that when am older, I will go the other clinic*” (P8).

Some also assumed that their caregivers were informed about the move to the adult clinic because the caregivers led and accompanied them to the adult clinic, and caregivers would not have taken this action without authorization from the HCP.

“*Maybe they informed my mother but as for me they did not inform me, so I just moved and joined because I didn’t even know about the sickness*” (P4)

#### Coordination of care

The sub-theme about coordination of care emerged when participants were asked about the transition process. When asked if they officially met with the HCP in the adult clinic prior to the transition all participants answered in the negative. Some also said that they never knew the adult HCP until they arrived at the adult clinic:

“*No, no, no, I didn’t get the opportunity to meet them until they brought me here finally*” (P3)

“*Ooh no oo, I met them the day my grandmum brought me*” (P8)

“*No, I didn’t know them before coming here (the adult clinic)*” (P10)

### Theme 2: factors facilitating the transition experience

This over-riding theme organizes the various features that participants identified as assisting the transition process. These features were captured by sub-themes about the child-specific factors and the family/societal supports participants identified that served to enhance their transition process.

#### Child-specific factors

This sub-theme code was based on the adolescent’s health-seeking and maintenance behaviors during transition. These self-care behaviors facilitated the child’s transition, displaying their level of maturity and evidence of maintenance behaviors. These behaviors included adherence to therapy as instructed, keeping clinic appointments, self-care management, and autonomy with care. These positive qualities made it easier for them to transition to adult care. When asked about their medication adherence and clinic appointments, the adolescents all responded that they took their medication with little or no assistance. Most went to the clinic by themselves to take their ART. Here are two sample responses:

*“I take my medications every evening. I take it by myself, so it wasn’t a problem when I moved to the adult clinic”* (P1)

*“I take it (medicine) by myself and sometimes too, my mother reminds me when she is taking her own. I take it in the morning and in the evening, I was taking it by myself long ago”* (P7)

Some participants were able to explain the various benefits they gain from taking their medicines regularly. The participants mentioned the support that ART provided in enhancing their health, which was beneficial to their smooth transition.

*“The time I stopped taking the medicines because my father asked me to stop, I fell sick frequently but from the time I started taking it again, I have been better, so I feel the medicine is helping me, so I understand the use of the medicine therefore I will continue to take and will not miss taking it here at the adult clinic”* (P6)

One participant who was initially non-adherent to his ART decided to be adherent after his HIV status was disclosed to him. The disclosure boosted his attitude towards adherence, which was perceived as key to a good transition. His mother’s explanation to him about the importance of the ART helped him to appreciate the effectiveness of the drug:

*“Yeah but I later stopped taking the medication for 3 years and later my mum told me and my diagnosis and I fell sick too and so I started to take it again” (*P4).

Keeping clinic appointments was another child-specific sub-theme that emerged from the data. The adolescents’ responses to questions asked about their attitudes towards clinic appointments, revealed that most of them did not have full control over their appointments. Most were coming to clinic accompanied by a caregiver. There were some instances when they came alone or their caregiver came alone to pick medications on their behalf. These adolescents did not perceive that they had any influence on whether they attended clinic appointments on their own or accompanied by an adult; it was influenced by the availability of a parent or caregiver as expressed in the following quotes:

*“I come by myself sometimes and sometimes with my mum or sometimes my mum will come for my medications for me*. *When she is traveling, she wants to ensure that my medicines are there before she goes, and also her workplace is close to the hospital”* (P2)

*“No, I always come with my mum because our house is very far from the hospital, and the route to town from where we live is not safe for me to come alone”* (P5)

#### Family support

This sub-theme explores the kind of support that the ALHIV receives from the family. Almost all (8 of 10) participants were living with their biological parent(s). In one case, an adolescent stayed with an elderly sibling because the parents had died from HIV, and in the other case she went to live with her grandparents when her mother died of HIV. The interview data also revealed that adolescents viewed their family support as enhancing the transition to adult care. They specified examples of this type of support, which included parents accompanying them to the adult clinic, encouraging them to adhere and not discontinue attending the clinic regardless of the various discomforts they experienced at the new clinic environment. Some of the participants expressed how supportive their family caregivers were with medication adherence:

“*I take it (the medicines) by myself but my sisters also wake me up to take the medicine when I forget and I didn’t take it before sleeping, she always emphasize that I take it before sleeping so that I do not fall sick*” (P6)

In addition to the family caregivers’ encouragement for taking the ART religiously, the participants also commented on the supportive behavior of their caregiver when accompanying the adolescent to the clinic on some occasions:

“*Sometimes my grand mum brings me to the hospital and sometimes too, my aunty, they always encourage me to attend the clinic regularly … especially at the new place (adult clinic) where many old people are here but when my aunt or grandmother brings me, I feel more secure*” (P8)

### Theme 3: challenges and coping mechanisms during transition

In contrast with facilitating factors in Theme 2, this theme focused on the challenges that adolescents perceived during the transition and how they coped with these challenges.

#### Transition-related challenges

It was observed that most of the ALHIV seemed to cope well with the transition process, once their HIV status was disclosed to them. They did express a variety of psychological problems they encountered at various times during the transition period. These problems included fear of death, keeping to oneself, loneliness, crying, and some level of stigma. One adolescent expressed being sad about living with HIV: *“I feel bad, I can’t express it but just know that I feel bad about it (HIV)”* (P5). One adolescent explained her ordeal after a class lesson on HIV:

*“…we were taught about HIV in class and the teacher mentioned that, with HIV you can die early and it made me worried and fearful. I came back home and cry the whole evening, my mum had also travel, so I was in the house alone and cry the whole time. Whenever I remember that I will die, am always afraid”* (P5).

Another participant explained how she keeps to herself to avoid problems:

*“I don’t have problems anywhere because I always keep to myself at school and after school, am always in my room”* (P6).

Some participants also perceived that stigma would be attached to their HIV status, hence did not disclose their status to family or HCPs until trust was evident:

*“…they have been asking me why I often go to the hospital and I told them that it is nothing, so they were guessing and said that, is it HIV and I said that no! (Participant shouted). They were mentioning other disease and also mentioned HIV but when they mentioned HIV my heart was beating and my stomach was doing somethings at once but I said no, it is not HIV”* (P1)

#### Coping with challenges at transition

This sub-theme captures how participants managed or adjusted to the challenges they encountered during the transition process. Playing was the main strategy they identified for coping:

*“Always, I take the younger ones as my siblings so the ones that are small, small, I take them and play with them at the clinic though I don’t know them”* (P3)

“*I always play with the children but here in the adult clinic you can’t play like that, everybody is ready to be called by the health care providers*” (P10)

Worthy of note, reading and exercise such as jogging, were often mentioned as ways to cope with transition challenges as well:

*“I sometimes use my mother’s phone to browse and read if it is not yet my turn, I even read about HIV and other interesting things on the net”* (P9)

### Theme 4: transition approach and suggestions for improvement

When adolescents were asked about how they perceived the actual process of transition to adult clinic, they also described the HCP’s role in the approach, their expectations for what the transition process should be, and suggestions for improving the approach. Four sub-themes were identified.

#### Perception of the actual transition process

Participants varied in their views concerning the actual experience of transitioning from the pediatric clinic to the adult clinic. Some felt it was a normal step in their growth and development, and others were very happy to make this transition. Most recounted the preparation and the entire process to be more sudden than they preferred. Some did not like how information was relayed to them, preferring themselves to be given the information rather than their caregiver being given most of the information. Still others unfortunately discovered their HIV status only after the transition to the adult clinic, which they described as very displeasing.

Most of the adolescents spoke about the similarities of the services provided at the pediatric clinic and services provided at the adult clinic. Most responses focused on their experiences with other patients in the adult clinic. Others expressed how they felt having been introduced into an environment with adults compared to the more familiar environment with children. These experiences are exemplified in the following quotes:

*“Oh, it is okay, since you are growing, you have no option than to join the adult clinic one day, but I feel happy sometimes because it tells me that I am alive and growing with this sickness”* (P8)

*“It is the same, the medicine is the same and how I take it too is the same”* (P3)

Participants did talk about perceived differences between the medications given at the adult clinic and pediatric clinic. In fact, one participant remarked that the medication at the adult clinic induced sleep, as evident by this quote:

*“I thought I will be oversleeping when taking the medicine from the adult clinic because my mom always sleeps when she takes the medicine…but I realized that when I sleep early I wake early and when I sleep late I wake late just like any other person”* (P5)

Some adolescents also expressed concerns about their ability or inability to make friends at the adult clinic due to the presence of so many adults:

*“The people are elderly, they may think that you don’t respect or something, but the doctors and nurses are my friends oo”* (P 8)

*“When I come to the clinic am quiet and just wait for my turn oo, I don’t play or talk to the other patients because they are not my friend, I don’t know them too and they are old too so I just stay in my lane”* (P10)

In contrast, some were happy to join the adult clinic:

*“I was happy that I moved to join the adult clinic because I was not okay when I was in the children clinic because all my mates were all kids, so I don’t have anyone to chat with so am alone here but this year, I have friends to talk with in the adult clinic”* (P 3)

#### Perceptions about the HCPs

When asked about transitioning to the adult clinic, most participants mentioned their perceptions about HCPs in both the pediatric and adult clinics. The HCPs provided much positive support for these adolescents. Two examples of their responses are expressed here:

“*The doctors and the nurses are my friends, but I don’t have any friends among the patients because I don’t know the other patients, so I don’t talk to them”* (P6)

“*They (HCPs) are nice, some of them even play with you and make you laugh”* (P10)

#### Perceived benefits of the transition

This sub-theme captured benefits the adolescents described when asked about their transition experience. For most of the adolescents, there was little difference between being at the pediatric and adult clinic. The most frequently mentioned benefit was the learning experience from the adult patients at the clinic. Also frequently mentioned was their sense of the assurance of life if they continue taking their medication. The adolescents who clearly identified these benefits wanted to remain in the adult clinic. Some of the benefits are captured below:

*“I feel normal, I also realize that, the way I used to be afraid that I will die, I will die, has reduce, I don’t know why but it has reduce, maybe too, because I am growing up”* (P9)

*“Oh it’s okay, since you are growing, you have no option than to join the adult clinic one day, but I feel happy sometimes because it tells me that I am alive and growing with this sickness”* (P8)

*“I feel normal, they don’t make noise like the children clinic children”* (P9)

*“We should not be separated from the adults… I learn from the adults more on the disease”* (P1)

#### Expectations about the transition process

Most participants described what they expected for the transition process including the preparation, verbal/written information provided to them, their involvement in the process and the opportunity to meet the adult clinic care team prior to transitioning. Some adolescents expected some planned teachings before the transition, as expressed by this quote:

*“I thought they will set a meeting for us to teach us more and maybe set exams for us to write, then when you pass the exams, then you qualify to go to the adult clinic just like we do at school because the nurse used to say that you need to know everything before they let you go (laughing)”* (P10)

Some also expressed that the transition was so abrupt but they had no option other than to oblige.

*“They told myself and my mum about the transition but I didn’t know that it’s going to be that day that I finally came to the adult clinic but I just have to obey”* (P8)

Others also stated that more verbal and written instructions should be required about the transition process, as elaborated in these quotes:

*“They (HCPs) can give us some posters just like the one on the wall (a poster describing HIV prevention) about when or how we will move to the adult clinic then you can even read it at home” (*P8)

*“I was thinking that the child will rather be given more information about going to the adult clinic but in my case my mother was told everything without me (sad face)”* (P4)

Some adolescents expected more collaboration and integration from the HCPs during the transition process. One suggestion was exemplified by this quote:

*“The people there (the HCPs at the children clinic) can come and introduce me to the people here (HCPs at the adult clinic) so that it makes it easier”* (P6).

#### Suggestions for improvement

In the quest for better transitional care, recommendations were conveyed during their interviews in the hopes of enhancing the services they receive. Suggestions were offered for improving transitioning care/services at both the pediatric clinic and the adult clinic. Most participants were pleased with the services at the pediatric clinic. Only a few participants suggested ways to improve these services, with suggestions focused on more pre-transfer information for adolescents. One adolescent suggested that all adolescents be seen together, separated from the adults. Other suggestions are seen in the quotes below:

*“They should inform me, they should inform me rather, they should inform the child rather than the mother”* (P4)

*“My suggestion is that, if they will transfer someone to the other clinic, they must let the person be aware and must interview the person to be sure, the person should be fine*…*I wish that they give all of us the same date, so all my age mates will come together so that I have more friends”* (P3)

One participant suggested changes regarding laboratory tests as part of the preparation for transitioning. She expressed the following:

*“I think they should ask us to do viral load and others laboratory investigations, but I only did the viral load because we have other laboratory investigations that we usually do but maybe it wasn’t necessary at the time of transition”* (P5)

In contrast, another participant was required to get a viral load test result before transitioning. He.

expressed it this way:

*“...[HCP] also asked me, do I really take the medicine well, I said yes, then they ask me to do a viral load to check if it is really true, so I tested for the viral load and the animals (viruses) in me was reducing gradually, so they said I should keep it up so that I always take my medicine, I shouldn’t even leave a day and continue taking the medicine and not even miss my medicine for one day”* (P3)

The adolescents expressed appreciation for the services and for their HCPs. When probed for possible improvements, they offered suggestions to make their exit from pediatric clinic more comfortable and satisfying. Some suggested Saturday services and others suggested adolescent services to bring all adolescents together for care. They expressed these various perceptions:

*“Sometimes the date they will give me will not be the same date that they will give to my age mates, that is what is worrying me, so I wish that they all of us the same date, so all my age mates will come together so that I have more friends*” (P3)

*“I think they should hold the clinic on Saturdays so that I don’t miss class and lessons and my mates and teachers will not be questioning me about the sickness. Maybe other people too will like the Saturday one because some will go to school and some will go to work. They can also give one date to all the young ones so that we come together then we come together*” (P5)

Another important suggestion by many participants worth noting was the request that HCPs consider establishing a separate clinic especially for adolescents. There were many similar responses, but the relevance of this recommendation is summarized by this participant’s comment:

*“Maybe they can bring all my age mates together so that we come as a group but they give us different dates but even today the nurse was telling me that, they will start letting all my age group to come on Thursday so that we come on Thursday and the elderly people will come another day so maybe next time I may come on Thursday and not Friday. They can even let us come on Saturday so that you sign exeat [permission] to come on weekends but the week day one is difficult because you have to travel to this place, so sometimes my grandmother comes for it for me, or they give us plenty medicines for one term”* (P8)

## Discussion

While HIV and ART services are available and accessible for ALHIV, there is no structured guide for adolescent to transition from pediatric to adult care. This study examined the experiences of ALHIV who transitioned from pediatric to adult HIV care. Adolescent healthcare transition is a concept that ensures a planned and purposeful movement of adolescents from child-centered to adult-centered care. The components of any transition protocol should include planning and preparation for the transition, coordination and integration, and post-transition care.

### Preparing for transition with a structured protocol

A key finding of this study was the sudden preparation for transition, linked to the absence of a structured transition protocol. Most participants were not aware of the transition but their parent or caregiver had been informed. Most participants also did not know what to expect at the adult clinic. This finding was surprising because since 2014, experts have emphasized the importance of transition counselling sessions to set adolescent-focused objectives for transitioning [16, 20] based on WHO recommendations for a structured transition from pediatric clinic to the adult clinic [10, 21]. The absence of a structured transition protocol with individualized objectives as seen in this study leaves the adolescent patient with inadequate information as to why the transition is necessary, with a sudden knowledge of their disease, and with little support for adapting to the process [22].

Age is widely reported as the main impetus for transferring from the children’s HIV clinic [9, 23] yet Tepper and colleagues [8] recommend an individualized approach to elicit the adolescent’s readiness for transition to adult clinic. Our findings support this recommendation. The main factor that informed the transfer in our study was age 13 years, but our participants were transferred at different ages and under different conditions and based on their responses, most ALHIV were informed that the reason for their transfer was being too old to go for their ARV at the children’s clinic and lack of an intermediary clinic to serve the needs of adolescents.

Although there was no structured assessment tool for readiness and preparation for ALHIV transition, these two assessments were indirectly taken into consideration in our sample, based on the ALHIV’s ability to honor clinic appointments and remain adherent to their ARV regimen. These two important self-management skills are key assessments indicative of ALHIV readiness for the transition. Irrespective of the age at which the adolescent is transitioning, Soeters and colleagues [16] recommend using a comprehensive readiness assessment tool for an individualized approach to transitioning that will ensure adolescent readiness.

In addition to assessments for readiness and preparation, some participants were told to undergo viral load testing to document their disease progression before joining the adult clinic. This is in line with Centers for Disease Control and Prevention recommendations on transition, advocating for education on transition and empowerment around self-management, which should commence with the adolescent early in care and include a package of individualized and age-appropriate techniques such that each adolescent will be totally prepared for the transition [24].

### Coordinating and integrating the transition

Our findings also revealed a poor level of transition care coordination and integration between the pediatric and adult clinics. There was no scheduled meeting with the HCP in the adult clinic, and in the few instances where the adolescents were accompanied to the adult clinic, limited communication took place. According to Kung [25], transferring an adolescent in such manner implies that he or she is unprepared for going to what is likely to be a judgmental, depersonalized and overburdened clinic environment. Although the participants mentioned having good relationships with the HCPs, there was evidence of perceived ineffective bonding that could influence their experience of separation from the pediatric clinic to join the adult clinic. In mentioning that the HCP played with them and was friendly towards them, there was no strong feeling expressed about the relationship that hindered or delayed the transition. Although it felt sudden, they left with ease and expressed no form of protest against the transition. This finding is inconsistent with other studies in which the adolescents developed relationships with their HCPs, often seeing them as members of their family and were unwilling to move to the adult clinic [26, 27].

Aside from a friendly relationship with their HCPs, adolescents in this study mentioned their inability to make friends even though they played occasionally when the opportunity presented itself in the pediatric clinic. This implied that they had no peer relationships at the pediatric clinic, which may help to explain why they were not reluctant to move to the adult clinic despite inadequate preparation. Our findings are in contrast to a study where ALHIV viewed transitioning to adult clinics as a hostile process that disconnected their relationships with friends and HCPs under protest [12].

Parents and adult family caregivers were integrated into the transition process to some extent according to participants in our study. It was clear that most of the adults were given much information than the adolescents themselves were given. For almost all participants, the caregiver happened to receive the firsthand information about transitioning and then served as a mediator between the ALHIV and the HCPs. This was consistent with a study of caregivers who acted as facilitators to the transition process [28]. This protocol may be advantageous to promote caregiver support during the transition, however, it denies the adolescent any direct information from the HCP [29].

In our sample, the ALHIVs made the transition easier by their willingness and the acceptance of the move to the adult clinic without protest. The ease in transition was also evident by their perception that ARV maintenance instructions eased their transition process. However, our findings contradict other studies where the adolescents found the entire transition process to be more challenging [9, 22]. Despite the lack of a formal transition process, our participants expressed feeling in control and responsible for their healthcare transition. The adolescents’ responses indicated that they were committed to care and felt even more responsible compared to when they were in the pediatric clinic. This finding is in contrast to a study reporting poor adolescent transition linked with loss to follow-up, poor retention in care, and poor adherence to ART [9].

While the adolescents talked about support from family and HCPs, there was little discussion about support from other sources, such as the school system. Because the school permitted adolescents to only leave school to attend clinic appointments, the adolescents gave false excuses to hide their HIV status in order to obtain permission. It is unclear if the school would still accommodate an excused absence should their status be known and there are no special services rendered within the school domain for ALHIV. Our findings are consistent with a study by Abubakar and colleagues [30] that also described a school system unresponsive to the needs of ALHIV, with no support for the ALHIV beyond the family and the clinic. HIV status of the adolescent is often kept secret from the community due to fear of stigmatization, making it difficult to determine if indeed the ALHIV faces stigma at school or in the community. However, lack of knowledge about HIV in school curricula is a known risk factor for stigmatization. Our findings are similar to a study by Mensah [31] who documented that adolescents keep their HIV status secret from the community as well as some family members to prevent stigma, rejection and discrimination. Apart from their perceived stigma making them secretive about their diagnosis, all the adolescents in our study responded that no form of stigma or discrimination had been levelled against them.

Although thought to be inadequate, the parental and clinic support systems in place for these adolescents was perceived as highly satisfactory. This finding was similarly documented by a previous study [32]. Caregiver support was available, often in the form of constant reminders to take their ART. Healthcare was readily accessible, they had enough medication, and ART was provided at no cost to them. These findings support findings of other researchers who reported caregiver support as one of the main facilitators for adherence to ART [31, 33].

In summary, we found inadequate preparation for transition, poor communication between the pediatric and adult clinic HCPs, ineffective assessment of transition readiness, lack of a transition plan and an unclear transition process. In addition to these transition challenges, there was poor attention to level of autonomy and little societal support encountered by the ALHIV. This is affirmed by other studies globally where similar findings were reported. Others have also reported challenges at transition that include lack of a structured healthcare transition, lack of communication between the pediatric and adult HCPs, mental health problems, strong attachment to the adolescent’s pediatric clinic HCP, privacy/disclosure issues, negative perceptions of the adult clinic HCPs, and stigma [17, 25].

### Coping with transition

In this study, browsing the internet, reading, and playing were the stated means of coping with transition distress. This is similar to what has been reported in some studies, yet other studies found use of substances mentioned as a coping mechanism. None of the participants in this study mentioned substance use as a means of coping. Some previous studies stated that coping strategies used by ALHIV varied greatly, from resignation, self-calming and distraction, to less used techniques such as resorting to alcohol and substance abuse [7, 34]. Furthermore, the adolescents in the current study recognized and coped well with their HIV status even though they expressed feelings of psychological trauma including hurtfulness, discomfort, sadness, guilt and fear of death. This was similar to a previous study where the participants also expressed fear, worry, pain, and grief [35].

Amidst the several challenges voiced by the ALHIV, they perceived the transition as a learning opportunity that must be experienced. They felt okay joining the adult clinic without any signs of agitation. They expressed excitement about the opportunity to learn new information about their status from the adults. This implied that the clinic itself did not provide sufficient information about their HIV status, which may account for the inadequate knowledge about HIV among the adolescents. The major challenge expressed by the adolescents was the inability to make friends at the adult clinic. While they talked about their inability to go to clinic on school days it did not present as a challenging issue because parents and caretakers were able to acquire the medication on their behalf. This is in contrast with a study in which the adolescents were less satisfied with the transition process and felt pushed into the adult care service without sufficient preparation [36]. Our participants also mentioned having good relationships with their HCPs even though most of them were not attached to the pediatric HCP, which made for a smoother transition. The results are similar to a previous study in which ALHIVs who were more attached to their pediatric HCPs struggled to transition while those who were less attached or had no attachment experienced an easy transition [37]. The participants in this study perceived no significant change in the care delivery at the adult clinic, but some highlighted that the pediatric clinic was more playful, with children playing around, in contrast with the adult clinic where patients were seen in the queue waiting for their turn to see the consultant. Adult HIV clinics are often more formal and business like in approach and nature [5].

### Post-transition care

Despite the challenges, the adolescents provided suggestions to improve transition care delivery. Most suggested that clinic days should be specific for age mates, in direct response to the challenge of not being able to make friends at the clinic. By having the same clinic days, they can meet each other more frequently, become friends, and learn from these friends as well. It is not clear however, if the benefits of learning from age-matched peers are all positive. Older patients might provide information and survival tips based on their experience. Also, without interacting with older patients, the ALHIV may gradually lose sight of living a healthy adult life. Previous studies that recommended transition clinics for adolescents documented that inadequate support and poor preparation for the transition to adult clinic care can jeopardize the outcome of the transition [7, 13]. They also caution about the danger of losing sight of the benefits of interacting with other adolescents, which provides them with strength, hope and encouragement because they know they were not alone. Seeing other adolescents going through similar experiences can reduce the feelings of depression and loneliness and provide the adolescents with a sense of belongingness [31, 35].

### Strength and limitations

A qualitative study approach provided the opportunity to explore the issues of ALHIV transitioning from pediatric to adult car in more depth than would be applicable in quantitative studies. Although this may affect generalizability to all ALHIV, qualitative studies are likely to provide direction on what to explore or consider in a subsequent quantitative approach. The research involved the patient’s own experiences of events, which was subjective. However, the amount of uniformity in responses is convincing and forms a source of study credibility. This study was not designed to measure the association between facilitators and challenges or the direct components of the transition process. Because most of our participants were living with a parent, future studies should focus on ALHIV who are not living with parents.

In this study, recall bias was possible because the data are based on the retrospective experiences of the participants, and some may have difficulties in recalling vividly exactly the issue they experienced. Although the participants and their parents were assured of confidentiality, some of the parents might have advised the participants with respect to their expressions, which may have led to the participants suppressing some vital information that would have enriched the results of the research. However, the researcher used open-ended questions and additional probes to elicit more detailed information from the participants.

## Conclusion

This study revealed some weaknesses in health services with regards to the healthcare transition process of ALHIV, as the unstructured healthcare transition process that is currently the case, can disrupt the continuity of care for ALHIV. Based on our findings, the age of the adolescent was not as relevant as the crucial need for a well-planned transition process with greater involvement of the ALHIV and collaborative efforts from relevant stakeholders such as HCPs and family caregivers. The findings from this study indicate a need for the National HIV/AIDS/STI Control Program to strengthen the health system by facilitating context-specific policy guidelines informed by WHO recommendations to address the training needs of HCPs towards effective healthcare transition practices of ALHIV. In addition, the result indicate that further research is essential in developing an intervention model with respect to the transition process that is appropriate to support ALHIV in their healthcare transition. Future studies should include interviews with HCPs to understand their experiences of transferring adolescents from pediatric HIV clinics to adult clinics. Other key informants could also be interviewed, such as the Director of the National HIV/AIDS/STI Control Program, to better understand the policy implications of transitioning the ALHIV from pediatric services to adult clinic services.

## Data Availability

The datasets generated and/or analyzed during the current study are available from the corresponding author upon request.

## Abbreviations

AIDS: Acquired Immune Deficiency Syndrome
ALHIV: Adolescents living with HIV
ART: Anti-Retroviral Therapy
ARV: Antiretroviral
GHS: Ghana Health Service
HCP: Healthcare Providers
HIV: Human Immunodeficiency Virus
UNAIDS: United Nations Program on HIV and AIDS
WHO: World Health Organization
